# Schnelle Hilfen für Betroffene von akuter Gewalt – aktuelle Versorgung in Traumaambulanzen in Deutschland

**DOI:** 10.1007/s00115-024-01676-8

**Published:** 2024-06-04

**Authors:** Marc Giesmann, Isabella Flatten-Whitehead, Lina Specht, Jörg M. Fegert, Julia Schellong, Miriam Rassenhofer, Ingo Schäfer

**Affiliations:** 1 Klinik für Psychiatrie und Psychotherapie, Universitätsklinikum Hamburg-Eppendorf, Hamburg, Deutschland; 2 Klinik für Kinder- und Jugendpsychiatrie/Psychotherapie, Universitätsklinikum Ulm, Ulm, Deutschland; 3 Klinik und Poliklinik für Psychotherapie und Psychosomatik, Universitätsklinikum Carl Gustav Carus, Dresden, Deutschland; 4 Klinik für Psychiatrie und Psychotherapie, Universitätsklinikum Hamburg-Eppendorf, Martinistr. 52, 20246 Hamburg, Deutschland

**Keywords:** Trauma, Posttraumatische Belastungsstörung, Versorgung, Therapie, Ambulanz, Trauma, Posttraumatic stress disorder, Care, Treatment, Outpatient

## Abstract

**Hintergrund:**

Hilfen nach akuter Gewalt wurden bislang durch das Opferentschädigungsgesetz (OEG) geregelt. Zu Beginn des laufenden Jahres wurde es durch das Sozialgesetzbuch XIV (SGB XIV) abgelöst. Durch das SGB XIV werden neue Gruppen von Leistungsberechtigten definiert, Traumaambulanzen müssen flächendeckend vorgehalten werden und es wurden verbindliche Kriterien für die Qualität der Versorgung festgelegt. Die vorliegende Untersuchung hatte zum Ziel, den aktuellen Stand der Versorgung in Traumaambulanzen nach den Vorgaben des neuen SGB XIV abzubilden. In Bezug auf neue Leistungsberechtigte wurde exemplarisch der Stand der Angebote für Betroffene von Menschenhandel erfasst.

**Methodik:**

Ambulanzen, die Schnelle Hilfe nach dem OEG bzw. SGB XIV anbieten, wurden zu strukturellen und inhaltlichen Aspekten ihrer Arbeit befragt. Dabei kam ein Online-Survey zum Einsatz, der aus 10 thematischen Modulen bestand. Es konnten Daten von insgesamt *N* = 110 Ambulanzen erhalten werden (Rücklaufquote 50 %).

**Ergebnisse:**

Die teilnehmenden Ambulanzen berichteten eine breite Spannweite in Bezug auf die Anzahl der dort Beschäftigten und die Anzahl der Ratsuchenden. Bei einem Teil der Ambulanzen zeigten sich Defizite in Bezug auf strukturelle Aspekte wie die Wartezeit bis zum Erstgespräch und spezifische traumatherapeutische Fortbildungen beim Personal. In Bezug auf den Umgang mit Betroffenen von Menschenhandel bestand beim überwiegenden Teil der Ambulanzen Unsicherheit.

**Diskussion:**

Traumaambulanzen scheinen ihre Zielpopulation zu erreichen und angemessene Angebote zu deren Versorgung vorzuhalten. Allerdings weist ein bedeutsamer Teil der Ambulanzen Nachbesserungsbedarf auf, um die Qualitätskriterien des SGB XIV zu erfüllen und neue Gruppen von Leistungsberechtigten angemessen zu versorgen.

## Abstract (en)

**Background:**

Assistance following acute violence was previously regulated by the Victim Compensation Act (OEG). At the beginning of the current year it was replaced by the Social Code XIV (SGB XIV). The SGB XIV defines new groups of beneficiaries, outpatient trauma clinics must be provided nationwide and binding criteria for the quality of care were established. The aim of this study was to map the current status of care in outpatient trauma clinics in accordance with the requirements of the new SGB XIV. With respect to new beneficiaries, the status of services for victims of human trafficking was recorded as an example.

**Methods:**

Outpatient clinics that provide rapid assistance under the OEG or SGB XIV were surveyed on structural and content-related aspects of their work. An online survey consisting of 10 thematic modules was used. Data were obtained from a total of *N* = 110 outpatient clinics (response rate 50%).

**Results:**

The participating outpatient clinics reported a wide range in terms of the number of staff and the number of people seeking counselling. Some of the outpatient clinics reported deficits with respect to structural aspects, such as the waiting time for the initial consultation and specific training in trauma treatment for staff. The majority of outpatient clinics were uncertain about how to deal with victims of human trafficking.

**Discussion:**

Outpatient trauma clinics appear to reach their target population and provide appropriate services for their care; however, a significant number of outpatient clinics need to make improvements in order to fulfil the quality criteria of SGB XIV and provide adequate care to new groups of beneficiaries.

Bei Menschen, die Opfer einer Gewalttat geworden sind, können frühzeitige Interventionen Belastungen reduzieren und das Risiko von Traumafolgestörungen senken. Entsprechende Hilfen wurden bislang auf Basis des Opferentschädigungsgesetzes (OEG) in Traumaambulanzen angeboten. Mit dem Sozialgesetzbuch XIV (SGB XIV) werden sie gesetzlich verankert und müssen sich neben verpflichtenden Rahmenbedingungen auf neue Gruppen von Leistungsberechtigten einstellen. Bislang ist unklar, inwieweit die existierenden Traumaambulanzen diesen Vorgaben entsprechen.

Das Opferentschädigungsgesetz (OEG) berechtigte Betroffene von Gewalt in Deutschland Soziale Entschädigung, z. B. in Form von Renten und Ausgleichszahlungen, in Anspruch zu nehmen. Dazu zählten auch beratende Gespräche und psychotherapeutische Behandlung als Leistungen, unter anderem, um einer Chronifizierung möglicher Traumafolgen frühestmöglich entgegenzuwirken.

Diese Leistungen wurden ursprünglich in Nordrhein-Westphalen modellhaft in sog. OEG-Traumaambulanzen erbracht. Dafür schlossen Sie einen Vertrag mit den zuständigen Versorgungsämtern, die die dazugehörige Finanzierung stellten [z.B. 5, 16]. Das Konzept wurde im Verlauf von verschiedenen anderen Bundesländern übernommen und mehrfach positiv evaluiert (z. B. [2, 9, 14]). Allerdings bestand bislang keine Verpflichtung OEG-Traumaambulanzen vorzuhalten, sodass sie nicht in allen Bundesländern verfügbar waren.

Das neue Sozialgesetzbuch XIV (SGB XIV) löst das OEG ab und trat am 01.01.2024 vollständig in Kraft. Es erkennt weitere Formen von Gewalt als Entschädigungsgrund an, mit neuen Gruppen von Leistungsberechtigten. Dies betrifft z. B. Personen, die durch Menschenhandel, Nachstellung, erhebliche Vernachlässigung im Kindesalter und Kinderpornographie geschädigt wurden. Staatsbürgerschaft oder Aufenthaltsstatus, so wird im SGB XIV betont, beeinflussen das Anrecht auf Soziale Entschädigung prinzipiell nicht. Auch die vormals OEG-Traumaambulanzen genannten Institutionen sind nun als Teil der sog. *Schnellen Hilfen* bundesweit festgeschrieben.

Der Gesetzgeber legt Qualitätskriterien für Traumaambulanzen durch die *Traumaambulanz-Verordnung* (TAV; [3]) fest. Sie bestimmt unter anderem die (Mindest‑)Qualifikation der dort Tätigen (§§ 3–5 TAV), Mindestzeiträume für telefonische und persönliche Erreichbarkeit der Traumaambulanzen und die maximale Dauer zwischen Kontaktaufnahme und Erstgespräch (§ 8 TAV). Auch sollen Betroffene das Geschlecht der Therapeut:in bestimmen dürfen und vor einem Wechsel der behandelnden Person geschützt werden (§ 2 TAV). Weiter verpflichtet die TAV die Ambulanzen, sich aktiv mit anderen „*örtlich ansässigen Organisationen und Leistungserbringern“ *zu vernetzen, die Hilfs- und Unterstützungsangebote für Leistungsberechtigte bereitstellen. (§ 11 TAV). Die TAV stellt somit das gesetzliche Grundgerüst für die Rahmenbedingungen von Traumaambulanzen dar, während für die inhaltliche Arbeit mit Betroffenen von akuter Traumatisierung und Traumafolgestörungen die relevanten Leitlinien herangezogen werden können [4, 17].

Die vorliegende Untersuchung hatte zum Ziel, den aktuellen Stand der Versorgung in Traumaambulanzen vor dem Hintergrund der Vorgaben des neuen SGB XIV abzubilden. Dabei sollten potenzielle Verbesserungsbedarfe in Bezug auf eine adäquate Versorgung erfasst werden, sowohl in Bezug auf strukturelle als auch auf inhaltliche Aspekte der Arbeit in den Ambulanzen.

## Methodik

### Stichprobe

Es wurden *N* = 234 Traumaambulanzen zur Teilnahme an einem Online-Survey eingeladen, die einen gültigen Vertrag zur Abrechnung nach dem OEG bzw. *Sozialen Entschädigungsrecht (SER) *mit dem jeweiligen Bundesland hatten (Stand März 2023). Die Identifikation passender Traumaambulanzen basierte auf einer umfangreichen Recherche anhand entsprechender Verzeichnisse einschließlich der offiziellen Listen auf Webseiten der Bundesländer. Alle Einträge wurden in eine zentrale Datenbank überführt, durch weitere Daten angereichert und systematisch validiert. Dazu wurden die Webseiten der infrage kommenden Traumaambulanzen nach entsprechenden Informationen zur medizinischen bzw. psychotherapeutischen Behandlung nach dem OEG bzw. SER und dazu passenden Ansprechpartner:innen durchsucht.

### Erhebungsinstrument

Ein vollstrukturierter Onlinefragebogen wurde mithilfe von LimeSurvey 5 erstellt, der Fragen zu strukturellen Eigenschaften, Eckpunkten inhaltlicher Arbeit und grundlegenden Qualitätsstandards enthielt. Dabei wurde sich an Kriterien des SGB XIV bzw. der *Traumaambulanz-Verordnung* (TAV) orientiert, sodass auch die Versorgung neuer Leistungsberechtigter berücksichtigt wurde. Insgesamt wurden 37 Fragen in 10 thematisch zusammenhängenden Modulen gestellt.

Als Antwortformate wurden verschiedene Likert-Skalen eingesetzt. Dazu kamen Fragen zu geschätzten relativen Häufigkeiten (z. B. *In Prozent: Bei wie vielen der im letzten Jahr nach OEG behandelten Klient:innen wurde im Laufe der Behandlung in Ihrer Ambulanz eine PTBS diagnostiziert?*), Zeitintervallen (*Wie lange ist die durchschnittliche Zeit zwischen der ersten Kontaktaufnahme der Klient:in bis zum Ersttermin in Tagen?*) und absoluten, zählbaren Häufigkeiten (z. B. *Schätzen Sie: Wie viele von Menschenhandel Betroffene (z.* *B. Zwangsprostitution) waren im letzten Jahr bei Ihnen vorstellig?*). Ergänzend kamen qualitative Antwortformate in Form von Freitextfeldern zum Einsatz.

### Vorgehen bei der Datenerhebung

Der Survey wurde am 07.03.2023 eröffnet und personalisierte Einladungsmails an relevante Kontaktpersonen (zumeist Leitungspersonen) in den identifizierten Traumaambulanzen verschickt. Traumaambulanzen, die den Fragebogen 3 Wochen später noch nicht beantwortet hatten, erhielten eine Erinnerungsmail. Nach weiteren 3 Monaten erfolgten zusätzliche telefonische Kontaktaufnahmen mit den Ambulanzen, von denen bis dahin keine Rückmeldung erhalten wurde. Dabei wurden ggf. neue Ansprechpartner:innen erfragt, in die Datenbank eingepflegt und diese zum Survey eingeladen. Im Rahmen der telefonischen Kontaktaufnahmen stellte sich bei *n* = 7 Ambulanzen heraus, dass es sich bei ihnen nicht um eine OEG-Ambulanz handelte, d. h. kein gültiger Vertrag bestehe. Weitere *n* = 7 Ambulanzen gaben später, im Rahmen des Fragebogens an, dass kein OEG-Vertrag mit dem entsprechenden Bundesland bestehe. Die finale Stichprobe der kontaktierten OEG-Ambulanzen reduzierte sich dadurch auf *N* = 220.

## Ergebnisse

### Teilnehmende Ambulanzen

Von den *N* = 220 kontaktierten OEG-Traumaambulanzen beantworteten *n* = 110 den Fragebogen (Rücklaufquote 50,0 %). Dabei lag die Rücklaufquote in zwei Bundesländern unter 40 % (Bayern 37,0 %, Mecklenburg-Vorpommern 28,6 %), in 10 Bundesländern betrug sie 50 % oder mehr (Tab. 1).

**Tab. 1:** Teilnahmequote der kontaktierten Ambulanzen nach Bundesländern

Bundesland	Kontaktiert	Teilnehmende	
	*N*	*n*	(%)
Baden-Württemberg	7	5	(71,4)
Bayern	27	10	(37,0)
Berlin	3	3	(100)
Brandenburg	22	9	(40,9)
Bremen	6	3	(50,0)
Hamburg	3	2	(66,7)
Hessen	17	12	(70,6)
Mecklenburg-Vorpommern	14	4	(28,6)
Niedersachsen	29	14	(48,3)
Nordrhein-Westfalen	56	27	(48,2)
Rheinland-Pfalz	8	6	(75,0)
Saarland	2	1	(50,0)
Sachsen	4	3	(75,0)
Sachsen-Anhalt	2	1	(50,0)
Schleswig-Holstein	7	3	(42,9)
Thüringen	13	7	(53,8)
**Gesamt**	220	110	(50,0)

### Strukturelle Aspekte

Knapp drei Viertel (76,4 %) der Traumaambulanzen gaben an, dass es sich um Ambulanzen psychiatrischer Kliniken handele (*n* = 76) bzw. sie an andere, nicht näher bezeichnete Kliniken angebunden seien (*n* = 8). Weitere 7,3 % berichteten, Teil von Rehakliniken (*n* = 2) bzw. psychosomatischen Abteilungen (*n* = 6) zu sein, 11,8 % (*n* = 13), dass es sich um psychotherapeutische Ambulanzen handele. Schließlich berichteten 4,5 % (*n* = 3), dass es sich um psychiatrische Praxen handele sowie um Beratungsstellen (*n* = 2).

**Abb. 1:**
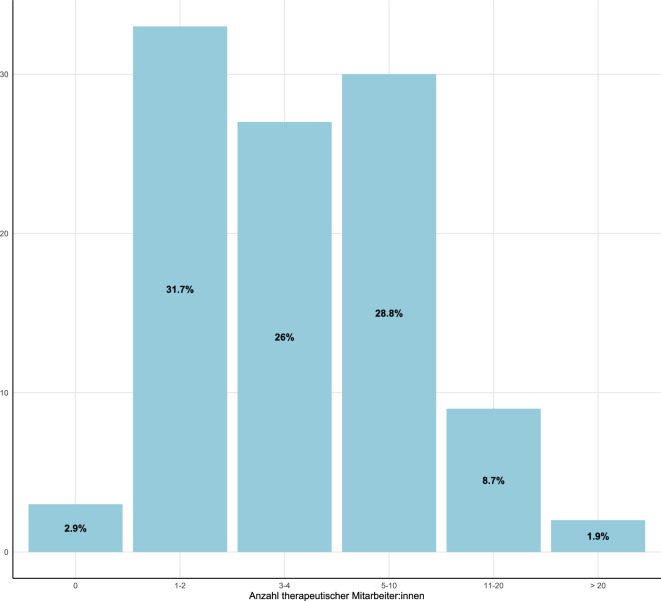
Anzahl therapeutischer Mitarbeiter:innen pro Ambulanz (*N* = 104)

Insgesamt 70,9 % (*n* = 78) der befragten Ambulanzen behandelten Erwachsene, 24,6 % (*n* = 27) Kinder und Jugendliche, 4,5 % (*n* = 5) boten Angebote für beide Gruppen gleichermaßen. In den meisten Fällen sind den Angaben zufolge 1 bis 2 (31,7 %, *n* = 33) bzw. 3 bis 4 (26,0 %, *n* = 27) therapeutische Mitarbeiter:innen neben der Leitung angestellt (Abb. 1).

Mehr als zwei Drittel (72,3 %, *n* = 85) der Ambulanzen gaben an, dass eine generelle 24-Stunden-Erreichbarkeit der Einrichtung bestehe, an die sie angebunden seien (z. B. über eine Notaufnahme). Die Mitarbeiter:innen der OEG-Ambulanzen waren dagegen nur in 5,5 % (*n* = 6) aller Fälle jederzeit persönlich erreichbar. In Randzeiten (vor 08:00 und nach 17:00 Uhr) könnten in 25,5 % (*n* = 28) der Ambulanzen auch telefonische und in 26,4 % (*n* = 29) persönliche Gespräche in Anspruch genommen werden.

Die Zeit bis zum Erstgespräch wurde von knapp der Hälfte der Ambulanzen (45,9 %, *n* = 50) mit durchschnittlich 1 bis 5 Tage angegeben. 39,4 % (*n* = 43) gaben an, dass mit durchschnittlich 6 bis 10 Tagen zu rechnen sei, bei 14,7 % der Institutionen (*n* = 16) betrage die Wartezeit mehr als 10 Tage. In *n* = 5 davon müsse sogar mit mehr als 15 Tagen gerechnet werden.

In 43,6 % (*n* = 48) der Traumaambulanzen werde mindestens alle 6 Wochen externe Supervision angeboten. 18,2 % Ambulanzen (*n* = 20) berichteten, dass es vorkommen könne, dass Klient:innen die therapeutische Person während der Therapie wechseln müssten. Das Geschlecht der Therapeut:in könne in 39,1 % (*n* = 43) Ambulanzen explizit durch die Klient:innen gewählt werden.

So gut wie alle befragten Institutionen meldeten zurück, dass psychosoziale Erstberatung (96,4 %, *n* = 106) und Psychotherapie (90,0 %, *n* = 99) bei ihnen angeboten werde. In etwa der Hälfte aller Fälle (48,2 %, *n* = 53) sei auch Medikationsverschreibung Teil des Angebots. In einigen Rückläufern wurde z. B. „Ausstellung von Arbeitsunfähigkeitsbescheinigungen“, „Leistungen der Jugendhilfe“, sowie „Schreiben für andere Stellen“ (z. B. für Rechtsanwälte und Sozialdienste) unter „sonstige Angebote“ ergänzt.

### Qualifikation der Mitarbeitenden

In den teilnehmenden Ambulanzen, so ergaben die Rückmeldungen (*N* = 104), seien über der Hälfte aller Leitungspositionen von Fachärzt:innen (FÄ) besetzt (55,8 %, *n* = 58), die restlichen 44,3 % (*n* = 46) mit Psychotherapeut:innen (PP). 65,4 % aller Leitungspersonen verfügten über eine anerkannte, traumaspezifische Zusatzqualifikation (*n* = 68). Dies betraf beide Professionen gleichermaßen (FÄ: 62,1 %, *n* = 36; PP: 69,6 %, *n* = 32). Eine Übersicht der jeweiligen Berufsgruppen findet sich in Tab. 2.

**Tab. 2:** Berufsgruppe der Ambulanzleitungen (*N* = 104)

Berufsgruppe	Zielgruppe			
	Erwachsene		Kinder und Jugendliche	
	*n*	%	*n*	%
FÄ Psychiatrie und Psychotherapie	29	15,4	3	1,0
FÄ K&J-Psychiatrie und Psychotherapie	–	–	13	2,9
FÄ Psychosom. Med. und Psychotherapie	13	1,9	–	–
Psycholog. Psychotherapeut:in	35	11,2	–	–
K&J-Psychotherapeut:in	–	–	11	1,9
Gesamt	77	28,8	27	5,8

*n* = 5 Traumaambulanzen behandelten sowohl Kinder und Jugendliche als auch Erwachsene. Davon besaß in *n* = 3 Fällen die Leitung eine Qualifikation im Erwachsenen-, in *n* = 2 Fällen im Kinder und Jugendbereich

Die Teilnehmenden gaben an, dass die meisten der therapeutisch arbeitenden Mitarbeiter:innen approbiert seien (75,8 %, *n* = 482), der kleinere Anteil (24,2 %, *n* = 154) befände sich in psychiatrischer oder psychotherapeutischer Weiterbildung. Mehr als jede:r zweite der *n* = 482 approbierten Mitarbeitenden (54,1 %) hatte den Angaben nach keine traumaspezifische Fortbildung. Von den *n* = 154 Mitarbeitenden in Ausbildung habe mit 76,0 % (*n* = 117) der weit überwiegende Teil diese erst begonnen (Tab. 3).

**Tab. 3:** Qualifikation der therapeutischen Mitarbeiter:innen (*N* = 104)

Berufsgruppe	Abgeschl. Weiterbildung zum FA bzw. psychol. PT				In fachärztl. bzw. psychoth. Weiterbildung				Gesamt	
	Mit traumaspez. Fortbildung		Ohne traumaspez. Fortbildung		Fortgeschritten		Begonnen			
	*n*	Anteil	*n*	Anteil	*n*	Anteil	*n*	Anteil	*n*	Anteil
Ärzt:in	69	10,8 %	71	11,2 %	14	2,2 %	54	8,5 %	208	32,7 %
Psycholog:in	124	19,5 %	133	20,9 %	19	3,0 %	57	9,0 %	333	52,4 %
Pädagog:in	23	3,6 %	48	7,5 %	3	0,5 %	5	0,8 %	79	12,4 %
Sonstige	5	0,8 %	9	1,4 %	1	0,2 %	1	0,2 %	16	2,6 %
Gesamt	221	34,7 %	261	41,0 %	37	5,9 %	117	18,5 %	–	–

### Anzahl der Klient:innen

In den vergangenen 2 bis 3 Jahren, so schätzten die Ambulanzen, nahmen im Median 14 erwachsene Klient:innen pro Jahr die entsprechenden Ambulanzen in Anspruch (*N* = 83, Range = 0–280). 28,9 % Ambulanzen (*n* = 24) gaben 0 bis 5, 16,9 % (*n* = 14) gaben 6 bis 10 Fälle pro Jahr an. 20,1 % der Ambulanzen (*n* = 20) sahen demnach durchschnittlich 11 bis 20 erwachsene Klient:innen, in 30,0 % (*n* = 25) der Rückmeldungen lagen die Fallzahlen darüber. Die vier größten Ambulanzen gaben an, durchschnittlich zwischen 100 und 280 erwachsene Klient:innen beraten zu haben.

Bei Ambulanzen, die auf Kinder und Jugendliche spezialisiert waren, lag der Median bei 5 Klient:innen (*N* = 32, Range = 0–150). Auch hier wurden in den meisten Ambulanzen bis zu 5 (53,1 %, *n* = 17), in 15,6 % der Ambulanzen 6 bis 10 (*n* = 5) und in weiteren 15,6 % (*n* = 5) durchschnittlich 11 bis 15 Kinder und Jugendliche in den letzten 2 bis 3 Jahren gesehen. Von einem vergleichbaren Teil der Ambulanzen (15,6 %, *n* = 5) wurden mehr als 20 Kinder und Jugendliche angegeben.

### Art der Kontaktaufnahme

Ratsuchende wenden sich den Angaben zufolge typischerweise eigenständig (96,3 %, *n* = 105) oder mithilfe von Vereinen für Opferhilfe (z. B. durch Weißer Ring e. V.) an die Ambulanzen (91,7 %, *n* = 100). Fachberatungsstellen (z. B. für sexuelle Gewalt) spielen bei 83,5 % (*n* = 91) der Ambulanzen als Zuweisende eine Rolle, gefolgt von Polizei (77,1 %, *n* = 84), Hausärzt:innen (77,1 %, *n* = 84) und Versorgungsämtern (65,1 %, *n* = 71). In den Freitextfeldern wurde ergänzt, dass auch kinder- und jugendspezifische Stellen (z. B. Jugendämter; 11,0 %, *n* = 12), Psychiater:innen und Psychotherapeut:innen (10,1 %, *n* = 11) sowie Nahestehende (Familie, Freunde oder Bekannte; 6,4 %, *n* = 7) die Ratsuchenden an entsprechende Traumaambulanzen verweisen würden.

### Diagnosen

Im Erwachsenenbereich (*N* = 83) wurde in den letzten Jahren am häufigsten (M = 51,3 %, SD = 27,1) die Hauptdiagnose „posttraumatische Belastungsstörung“ (PTBS) vergeben, gefolgt von „akute Belastungsreaktion/keine Störung“ (M = 21,3 %, SD = 25,5), „Depression“ (M = 15,0 %, SD = 18,6) und „sonstige Störungen“ (M = 10,9 %, SD = 19,7). Nur sehr selten wurde die „(partielle) dissoziative Identitätsstörung“ als Hauptdiagnose vergeben (M = 1,4 %, SD = 4,1).

Bei Kindern und Jugendlichen (*N* = 32) zeigte sich eine ähnliche Rangfolge: „PTBS“ (M = 56,2 %, SD = 30,4), gefolgt von „akute Belastungsreaktion/keine Störung“ (M = 23,4 %, SD = 29,5), „Depression“ (M = 7,3 %, SD = 11,1) und „sonstige Störungen“ (M = 7,3 %, SD = 16,1).

### Interventionen

**Abb. 2:**
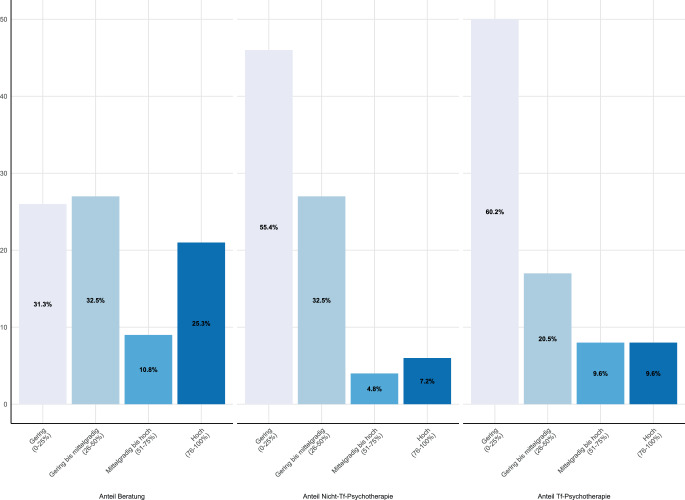
Verteilung der wahrgenommenen Angebote in Erwachsenenambulanzen (*N* = 83) in Quartilen

Ungefähr die Hälfte der Klient:innen erhielt den Schätzungen zufolge Beratungen (Erwachsene: M = 46,0 %, SD = 32,4, *N* = 83; Kinder und Jugendliche: M = 48,5 %, SD = 36,6, *N* = 32), die andere Hälfte Psychotherapien (Abb. 2 und 3). Therapieangebote ohne traumafokussierte Interventionen erhielt sowohl im Erwachsenen- (M = 27,4 %, SD = 25,5) als auch im Kinder- und Jugendalter (M = 25,3 %, SD = 26,6) ungefähr ein Viertel der Klient:innen, das restliche Viertel erhielt Psychotherapie mit traumafokussierter Intervention (Erwachsene: M = 26,6 %, SD = 28,8; Kinder und Jugendliche: 23,1 %, SD = 28,4).

**Abb. 3:**
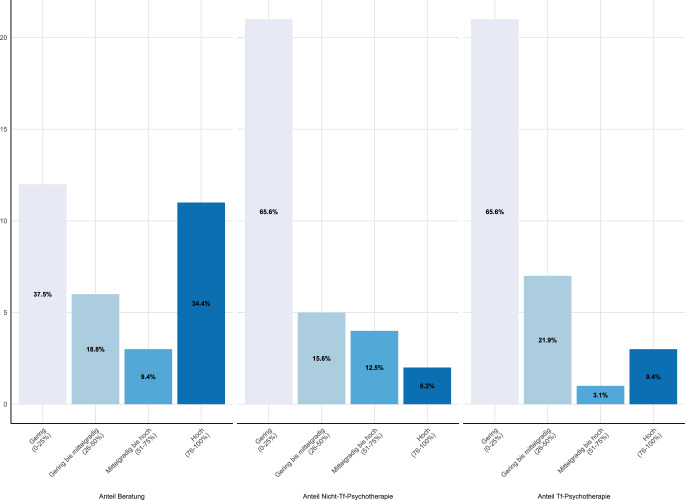
Verteilung der wahrgenommenen Angebote in Ambulanzen für Kinder und Jugendliche (*N* = 32) in Quartilen

### Versorgung von Betroffenen von Menschenhandel

Nur 11,5 % (*n* = 12) aller Traumaambulanzen berichteten, dass Betroffene von Menschenhandel, vor allem in kleinen Zahlen (≤ 4 Klient:innen im letzten Jahr), bei ihnen Rat suchten. Ein überwiegender Teil gab an, dass weder ihre Therapeut:innen noch andere Mitarbeiter:innen im Erkennen (87,5 %, *n* = 91), im traumasensiblen Umgang (86,5 %, *n* = 90) oder in der Behandlung Betroffener von Menschenhandel (87,5 %, *n* = 91) speziell geschult seien.

**Abb. 4:**
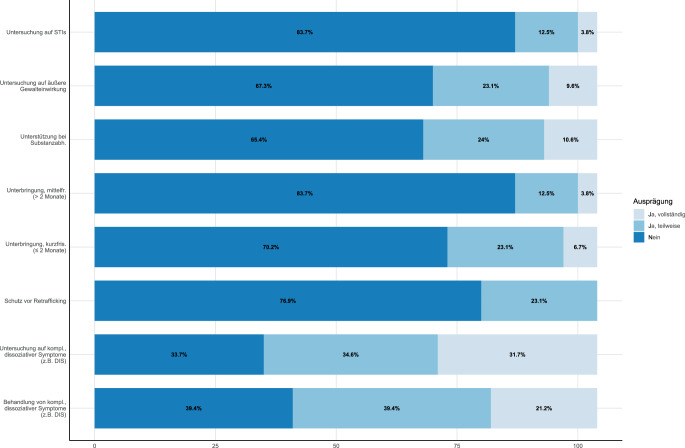
Implementierung traumasensibler Strategien zur Unterstützung Betroffener von Menschenhandel (*N* = 104). *STIs* „sexually transmitted infections“ (sexuell übertragbare Krankheiten)

Nur wenige Ambulanzen berichteten, traumasensible Strategien zur kurz- und mittelfristigen Unterbringung dieser Betroffenengruppe vorbereitet zu haben (Abb. 4). Ähnliche Inkonsistenzen zeigen sich mit Blick auf Schutz vor potenziellem Retrafficking, traumasensibler Untersuchung von Gewalteinwirkung, Substanzabhängigkeitserkennung und -substitution sowie Erkennung und Behandlung komplexer dissoziativer Symptome.

### Vernetzung

Im letzten Abschnitt wurden die Traumaambulanzen zu ihrer Vernetzung mit potenziell wichtigen anderen Stellen befragt.

**Abb. 5:**
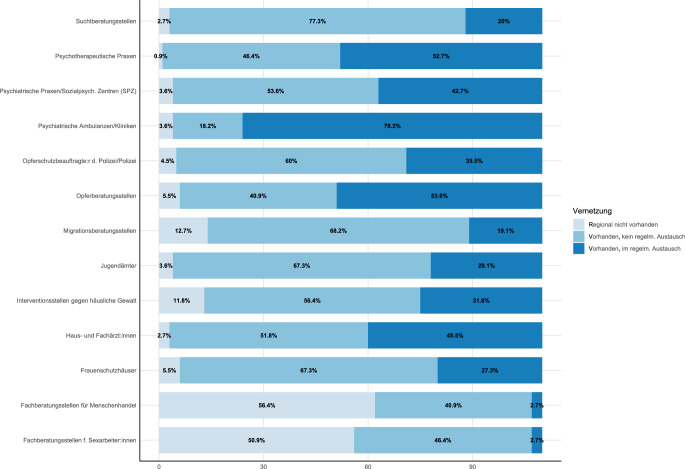
Vernetzung der Traumaambulanzen mit potenziell wichtigen anderen Stellen (*N* = 110)

Mit jeweils wenigen Ausnahmen wurde berichtet, dass das volle Spektrum der Anlaufstellen verfügbar sei (Abb. 5).

Regelmäßiger Austausch besteht laut Rückmeldungen oftmals mit psychiatrischen Ambulanzen (78,2 %), psychotherapeutischen Praxen (52,7 %), Haus- und Fachärzt:innen (45,5 %), psychiatrischen Praxen (42,7 %) sowie Opferberatungsstellen (53,6 %). Kontakte zu Angeboten, die sich mit Gruppen neuer Leistungsberechtigte befassen, bestehen demnach selten (Menschenhandel und Sexarbeit, je 2,7 %).

## Diskussion

Die vorliegende Untersuchung hatte zum Ziel, den aktuellen Stand der Versorgung in Traumaambulanzen nach OEG bzw. dem neuen Sozialen Entschädigungsrecht (SER) abzubilden. Die Hälfte aller kontaktierten Ambulanzen konnte für eine Teilnahme gewonnen werden. Auch in fast allen Bundesländern betrug die Rücklaufquote mindestens 50 %.

Der größte Teil aller teilnehmenden Ambulanzen (83,7 %) war an psychiatrische oder andere Kliniken angegliedert. Breite Leistungsangebote durch Klinikstrukturen, etwa Medikationsverschreibung oder 24-Stunden-Erreichbarkeit angegliederter Notaufnahmen, können Vorteile für Betroffene mit sich bringen. Dennoch könnte geprüft werden, Schnelle Hilfen vermehrt auch außerhalb von Klinikstrukturen anzubieten, z. B. um Versorgungslücken zu schließen oder Betroffene zu erreichen, die klinische Angebote als zu hochschwellig empfinden [7].

Die TAV definiert den Tätigkeitsrahmen der Traumaambulanzen (TA) und ergänzt Qualitätskriterien, die den Rahmen einer Beratung bzw. Behandlung bestimmen. So wird z. B. ein Rahmen und Zeitaufwand für bürokratische und prozessuale Unterstützung bei Antragsstellung definiert (§ 2 Abs. 1). Die §§ 3 bis 5 bestimmen Mindestqualifikationen von behandlerisch tätigen Mitarbeiter:innen der TA, die §§ 7 und 8 beschäftigen sich mit Vorgaben zu maximalen Fahrt- und Wartezeiten für Klient:innen. Zur Qualitätssicherung durch multiprofessionelle Vernetzung wird in § 11 aufgefordert.

Nach § 8 TAV müssen TA sicherstellen, dass Leistungsberechtigte spätestens 5 Werktage nach ihrer Kontaktaufnahme einen Termin erhalten. Vor diesem Hintergrund scheint die durchschnittliche Wartezeit zwischen Kontaktaufnahme und Erstgespräch oft zu lang zu sein. Weniger als die Hälfte der befragten Ambulanzen (45,9 %) gab an, dass der durchschnittliche Zeitraum zwischen Kontaktaufnahme und Erstgespräch wie vorgegeben 5 Tage betrage. Weitere 39,4 % gaben an, dass der durchschnittliche Zeitraum 10 Tagen betrage, was laut Rechtsverordnung dem Einzelfall vorbehalten sein sollte. In jeder 7. Ambulanz (14,5 %) muss den Daten nach sogar mit längerer Wartezeit gerechnet werden, in Einzelfällen sogar mit bis zu 6 Wochen. Dies könnte an zu geringer personeller Ausstattung oder internen strukturellen Barrieren liegen. So wurde bei den telefonischen Kontakten im Rahmen der Rekrutierung deutlich, dass nicht in allen Institutionen Klarheit in Bezug auf bestehende Angebote, Ansprechpartner bzw. Anmeldemodalitäten bestand. Eine Überprüfung interner Abläufe, mit Blick auf Wartezeiten und Informationsfluss, scheint besonders in Bezug auf Schnelle Hilfen nach akuter Traumatisierung empfehlenswert.

Die §§ 3 und 4 TAV sehen vor, dass therapeutische Mitarbeiter:innen in Ambulanzen als Berufsqualifikation prinzipiell eine abgeschlossene Fachärzt:innenausbildung oder eine Approbation als psychologische Psychotherapeut:in aufweisen müssen. Nach § 5 dürfen auch Personen in fortgeschrittener Weiterbildung (zwei Drittel der Weiterbildungszeit abgeschlossen) in die Behandlung einbezogen werden. Als traumaspezifische Qualifikation wird des Weiteren eine kurrikulare Fortbildung gefordert (Kurrikulum Psychotraumatherapie der Kammern bzw. der Deutschssprachigen Gesellschaft für Psychotraumatologie, DeGPT). Dies war bei einem Drittel der Ambulanzleitungen (34,6 %) und bei approbierten therapeutischen Mitarbeitenden in der Hälfte aller Fälle (54,1 %) nicht gegeben. Weiter zeigte sich, dass ein bedeutsamer Teil des Personals auch die geforderte grundständige Qualifikation nach § 6 TAV im Sinne einer „fortgeschrittenen“ Ausbildung zur Fachärzt:in bzw. psychologischen Psychotherapeut:in noch nicht erfüllte. Über alle Ambulanzen hinweg betrug der Anteil der Mitarbeitenden ohne ausreichende grundständige Ausbildung fast ein Fünftel (18,4 %).

Fast alle Ambulanzen berichteten, dass Ratsuchende sich selbst oder mithilfe von Vereinen für Opferhilfe an sie wenden würden. In Bezug auf Polizei und Hausärzt:innen berichtete ein Viertel der Ambulanzen, dass diese keine Rolle bei der Zuweisung spielten, Psychiater:innen und Psychotherapeut:innen wurden sogar nur von jeder 10. Ambulanz als Zuweisende genannt. In Bezug auf solche Zuweisungswege scheinen demnach noch deutliche Spielräume zu bestehen. So wurde die besondere Bedeutung von Hausärzt:innen für die Erkennung und fachspezifische Zuweisung traumatisierter Klient:innen in der Literatur immer wieder betont [10, 15]. Neben weiteren Vernetzungsaktivitäten nach § 11 TAV könnte deshalb die gezielte Kontaktpflege mit der primärärztlichen Versorgungsebene, aber auch mit Behörden hilfreich dabei sein, noch mehr Betroffene auf das Angebot der Traumaambulanzen aufmerksam zu machen.

Die befragten Traumaambulanzen schienen sowohl im Erwachsenen- als auch im Kinder- und Jugendbereich eher stärker belastete Personen zu erreichen. So erhielt etwas über die Hälfte der Klient:innen den Schätzungen zufolge die Diagnose posttraumatische Belastungsstörung (PTBS), für ein weiteres Viertel wurden affektive Störungen oder andere Diagnosen angenommen. Personen, bei denen keine psychische Störung diagnostiziert werden konnte, waren in der Minderheit. Etwa die Hälfte der Ratsuchenden erhielt psychotherapeutische Interventionen, die wiederum zu 50 % traumafokussiert waren, was einer leitliniengerechten Behandlung der PTBS entspricht [17] und einen höheren Anteil an fachgerechten Behandlungen nahelegen könnte als in der ambulanten psychotherapeutischen Regelversorgung [6].

Wie zu erwarten, traten Betroffene von Menschenhandel zum Zeitpunkt der Befragung, also vor dem Inkrafttreten des SGB XIV, fast in keiner Traumaambulanz auf. Mitarbeiter:innen wurden, trotz spezifischer Anforderungen zur Versorgung dieser Gruppe (z. B. [8, 11]), bisher kaum diesbezüglich fortgebildet. Obwohl Betroffene von Menschenhandel oftmals komplexere Störungen aufweisen [12, 13], halten nur wenige Ambulanzen traumasensible Strategien vor, um ihnen angemessen zu begegnen. Gerade mit Blick auf neue Leistungsberechtigte, die von komplexen Gewaltformen betroffen waren, scheint demnach Bedarf an Fortbildung und der Entwicklung angemessener Angebote (vgl. z. B. [1]) zu bestehen.

Weiter scheint ein erheblicher Bedarf an gezielter Vernetzung zwischen Traumaambulanzen und anderen Versorgungs- und Beratungsangeboten zu bestehen. Dabei liegt die Verantwortung zur Vernetzung mit „*ansässigen Organisationen und Leistungserbringern [..], die Hilfs- und Unterstützungsangebote für Leistungsberechtigte nach dem Vierzehnten Buch Sozialgesetzbuch bereitstellen*“ eindeutig bei den Traumaambulanzen (§ 11 TAV). Eine angemessene Vernetzung wird laut Rückmeldungen dadurch erschwert, dass nicht alle Angebote, etwa Fachberatungsstellen für Sexarbeiter:innen oder Betroffene von Menschenhandel, flächendeckend verfügbar sind. Die befragten Ambulanzen schienen jedoch auch mit potenziell verfügbaren Angeboten nicht immer in Kontakt zu stehen. So bestand ein Austausch mit Interventionsstellen gegen häusliche Gewalt, psychotherapeutischen Praxen oder Jugendämtern, laut den Rückmeldungen, nur in jeder 2. Ambulanz. Es scheint somit sinnvoll, Strategien für die Ambulanzen zu entwickeln, z. B. in Form einheitlicher Arbeitshilfen, um die Umsetzung lokaler Vernetzungsaktivitäten zu unterstützen.

Bei der Bewertung unserer Befunde müssen verschiedene Limitationen berücksichtigt werden. Da der Abschluss von Verträgen mit den Ambulanzen in der Verantwortung der Bundesländer liegt, existiert nach unserer Kenntnis kein einheitliches Ambulanzregister für ganz Deutschland. Es kann deshalb nicht ausgeschlossen werden, dass die Einladung zur Teilnahme an der Studie nicht alle Ambulanzen erreicht hat. Hinzu kommt, dass einzelne der kontaktierten Ambulanzen berichteten, nicht mehr im Rahmen des SER tätig zu sein. Auch erst seit kurzem aktive Ambulanzen könnten nicht erreicht worden sein. Weiter bleibt offen, wie zuverlässig die jeweiligen Einschätzungen getroffen wurden, etwa in Bezug auf die Anzahl der Fälle oder den Anteil verschiedener Diagnosen. Schließlich handelt es sich um einen Bereich, der sich aufgrund der Neuregelung der schnellen Hilfen für Gewaltbetroffene in stetiger Entwicklung befindet. Es ist davon auszugehen, dass sich durch den Abschluss neuer Verträge auch mit bereits aktiven Ambulanzen in den kommenden Jahren weitere Verbesserungen in Bezug auf die Struktur- und Prozessqualität in Traumaambulanzen ergeben. Dabei wäre ein einheitliches, bundesweites Monitoringsystem wünschenswert, um die entsprechenden Entwicklungen abzubilden und die Qualität der Versorgung in Traumaambulanzen langfristig sicherzustellen [7].

## Schlussfolgerung

Die existierenden Traumaambulanzen scheinen vor allem schwer belastete Menschen nach akuter Gewalt zu erreichen und angemessene Versorgungsangebote vorzuhalten. Verbesserungsbedarf zeigte sich bei einem Teil der Ambulanzen in Form zu langer Wartezeiten bis zum Erstgespräch. Für Klient:innen fehlte oftmals die Möglichkeit, das Geschlecht der Therapeut:in selbstbestimmt zu wählen, und weniger als die Hälfte der Ambulanzen berichtete, regelmäßig externe Supervision in Anspruch zu nehmen. Deutliche Defizite zeigten sich auch bei der Qualifikation des Personals. Dies betraf sowohl traumaspezifische Qualifikationen bei Leitungen wie Mitarbeitenden als auch den grundsätzlichen klinischen Ausbildungsstand bei einem Teil der Beschäftigten. In Bezug auf neue Gruppen von Leistungsberechtigten, exemplarisch gezeigt an Betroffenen von Menschenhandel, besteht weiterer Nachbesserungsbedarf in Form spezifischer Versorgungsstrategien. Schließlich sollten Anstrengungen unternommen werden, um Traumaambulanzen mit anderen relevanten Angeboten zu vernetzen.

## Fazit für die Praxis

- Im SGB XIV wurden Traumaambulanzen als Schnelle Hilfen gesetzlich verankert und neue Leistungsberechtigte, wie Betroffene von Menschenhandel, Nachstellung oder Vernachlässigung im Kindesalter, definiert.
- Strategien, um neue Leistungsberechtigte angemessen zu versorgen, scheinen noch nicht flächendeckend implementiert.
- Bei einem Teil der Ambulanzen besteht Nachbesserungsbedarf in Bezug auf Wartezeiten bis zum Ersttermin, regelmäßige Supervision der Therapeut:innen und traumaspezifische Zusatzqualifikationen.
- Eine weitere Vernetzung mit externen Angeboten sollte systematisch erfolgen.
- Ein bundesweites Monitoringsystem wäre wünschenswert, um die Qualität der Versorgung in Traumaambulanzen langfristig sicherzustellen.
