# Erratum zu: Schnelle Hilfen für Betroffene von akuter Gewalt – aktuelle Versorgung in Traumaambulanzen in Deutschland

**DOI:** 10.1007/s00115-024-01704-7

**Published:** 2024-06-27

**Authors:** Marc Giesmann, Isabella Flatten-Whitehead, Lina Specht, Jörg M. Fegert, Julia Schellong, Miriam Rassenhofer, Ingo Schäfer

**Affiliations:** 1 Klinik für Psychiatrie und Psychotherapie, Universitätsklinikum Hamburg-Eppendorf, Hamburg, Deutschland; 2 Klinik für Kinder- und Jugendpsychiatrie/Psychotherapie, Universitätsklinikum Ulm, Ulm, Deutschland; 3 Klinik und Poliklinik für Psychotherapie und Psychosomatik, Universitätsklinikum Carl Gustav Carus, Dresden, Deutschland; 4 Klinik für Psychiatrie und Psychotherapie, Universitätsklinikum Hamburg-Eppendorf, Martinistr. 52, 20246 Hamburg, Deutschland

**Erratum zu:**

**Nervenarzt 2024**

10.1007/s00115-024-01676-8

Der Abschnitt zur Finanzierung fehlte in diesem Artikel. Er hätte „Die Studie wurde vom Bundesministerium für Arbeit und Soziales gefördert (Va6-53406/16).“ lauten sollen.

Der Originalbeitrag wurde korrigiert.

